# Fractional Flow Reserve following Percutaneous Coronary Intervention

**DOI:** 10.1155/2020/7467943

**Published:** 2020-06-05

**Authors:** Udit Thakur, Nancy Khav, Andrea Comella, Michael Michail, Abdul R. Ihdayhid, Eric Poon, Stephen J. Nicholls, Brian Ko, Adam J. Brown

**Affiliations:** ^1^Monash Cardiovascular Research Centre, Monash University and MonashHeart, Monash Health, Clayton, VIC, Australia; ^2^Institute of Cardiovascular Science, University College London, London, UK; ^3^Department of Mechanical Engineering, The University of Melbourne, Parkville, Australia

## Abstract

Fractional flow reserve (FFR) is routinely used to determine lesion severity prior to percutaneous coronary intervention (PCI). However, there is an increasing recognition that FFR may also be useful following PCI to identify mechanisms leading to restenosis and the need for repeat revascularization. Post-PCI FFR is associated with the presence and severity of stent under-expansion and may help identify peri-stent-related complications. FFR pullback may also unmask other functionally significant lesions within the target vessel that were not appreciable on angiography. Recent studies have confirmed the prognostic utility of performing routine post-PCI FFR and suggest possible interventional targets that would improve stent durability. In this review, we detail the theoretical basis underlying post-PCI FFR, provide practical tips to facilitate measurement, and discuss the growing evidence supporting its use.

## 1. Introduction

Clinical outcomes following revascularization with percutaneous coronary intervention (PCI) have improved significantly over the last three decades, driven by advances in stent design, improved PCI techniques, and enhanced adjuvant pharmacotherapies [1, 2]. However, recurrent cardiovascular events remain an ongoing clinical concern, with around half of these either attributable to the previously stented segment or residual disease located within the target vessel [3].

Suboptimal procedural results are a potentially modifiable cause of repeat target lesion and/or vessel revascularization [4]. Even when an optimal angiographic outcome has been achieved following PCI, use of intravascular imaging reveals incomplete stent expansion, strut malapposition, geographical plaque miss, or stent edge dissection in approximately 50% of cases [4]. However, recent registry data confirm poor uptake of intravascular imaging, with this only being used in ∼5–15% of procedures [5, 6]. Several obstacles continue to limit uptake, including additional procedural time, operator comfort in image interpretation, and lack of reimbursement from healthcare funders.

Suboptimal stent deployment has also been associated with abnormal pressure gradients across the stented segment. This has led to increasing use of pressure wire interrogation following PCI [7]. A potential advantage of physiological parameters is the ability to identify residual untreated segments that may still produce myocardial ischaemia. Several studies have now shown that a low fractional flow reserve (FFR) in a vessel following PCI is associated with poor clinical outcomes [8–11]. These form part of the accumulating evidence which suggests that FFR following PCI has an important clinical role in the functional optimization of PCI.

In this review, we will discuss the physiological, theoretical, and clinical basis for the use of FFR for the assessment of stent deployment.

## 2. Rationale for Post-PCI FFR

### 2.1. Assessment of Stent Deployment

Lesions deemed satisfactory post-PCI using only angiography have significantly higher rates of suboptimal stent deployment [7, 8, 12]. Therefore, all patients with satisfactory PCI on angiographic assessment alone should have further assessment of the lesion to ensure adequate stent deployment. Suboptimal stent expansion is known to cause abnormal coronary conductance and nonphysiological blood flow patterns, leading to an increased risk of in-stent restenosis (ISR) and stent thrombosis (ST) [13]. Underdeployed stents are therefore known to be associated with an increased risk of major adverse cardiac events (MACEs) and target vessel failure (TVF) [4]. Lower post-PCI FFR measurements are correlated with suboptimal PCI results and predictive of future clinical outcomes [12, 14].

Low post-PCI FFR can predict stent under-expansion, malapposition, plaque prolapse, edge dissection, or residual disease [12, 15]. Many articles have demonstrated the ability of post-PCI FFR to predict suboptimal stent expansion, as assessed by either IVUS or OCT [7, 15, 16]. In 1999, Hanekamp et al. [16] enrolled 30 patients undergoing PCI in their study. Each stent was implanted at different inflation pressures, starting at 8 atm and increasing incrementally by 2 atm until 14 atm or until IVUS and FFR demonstrated an optimum result. In this study, the concordance between optimal stent deployment on IVUS criteria and FFR was 91%. In a further study, FFR < 0.8 following drug eluting stent (DES) implantation strongly correlated with subsequent TVF [7]. The same investigators also reported that stent length and diameter were all independent predictors for a greater pressure gradient after PCI within the left anterior descending artery (LAD). Similarly, a positive linear correlation (*r* = 0.70; *p* < 0.001) was found between post-PCI FFR and OCT-derived percentage area stenosis; with both measures shown to be predictors for suboptimal stent deployment [12]. In a cohort of patients with post-PCI FFR values ≤ 0.85, IVUS analysis revealed stent under-expansion in up to 74% of treated vessels [17]. Incomplete lesion coverage is the other main finding associated with a persistent pressure gradient after PCI [14]. Hence, post-PCI FFR is a surrogate for an optimal PCI result especially stent expansion.

### 2.2. Lower Endothelial Shear Stress as a Potential Trigger for In-Stent Restenosis and Stent Thrombosis

A low post-PCI FFR is known to be associated with low and/or oscillatory endothelial shear stress (ESS); which is known to propagate atherosclerosis, ISR, and consequent TVF [18]. Although the pathophysiology of ISR is multifactorial, changes in ESS have been hypothesized to play an important role [18]. ESS is the tangential stress due to the friction of blood flow on the endothelial surface and, in simple terms, is determined by arterial geometry, blood flow velocity, and viscosity. Human studies exploring the effects of ESS after bioresorbable stents have demonstrated that a low ESS promotes neointimal proliferation and excessive healing through increased inflammation, smooth muscle cell migration, and elastic lamina fragmentation [18, 19]. Many studies have reported an inverse relationship between ESS and neointimal thickness following bare metal stent (BMS) and DES implantation [20–22]. Low ESS also downregulates suppressors of cell growth and increases endothelial LDL particle accumulation, leading to atheroma progression. Low ESS causes endothelial activation of sterol regulatory element binding proteins (SREBPs), upregulating the expression of LDL receptor and cholesterol synthase genes [23]. In the context of systemic hyperlipidemia, this leads to an increased engagement and synthesis of LDL particles by endothelial cells promoting subendothelial accumulation [23].

**Figure 1:**
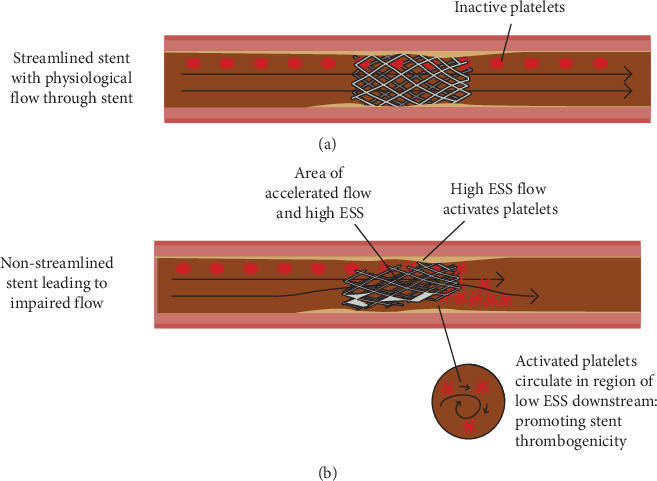
(a) Physiological flow through a well-positioned and expanded stent, facilitating re-endothelialization and inhibiting thrombus generation. (b) Poorly deployed stent leads to a region of accelerated flow and high ESS over the stenotic portion of the stent surface, which activates platelets to release vasoactive mediators including adenosine diphosphate. Adenosine phosphate along with downstream low ESS increases the local concentration of activated platelets, leading to increased stent thrombogenicity.

Furthermore, platelet-mediated prothrombotic effects of low ESS provide a mechanism for in-stent thrombus development. An underdeployed stent significantly alters arterial geometry, creating areas of high (with accelerated flow) and low ESS. These flow alterations are types of nonphysiological ESS that enhance platelet aggregation and thrombogenicity [24]. High ESS peaks over the stenotic portion of the stent surface activate platelets to release thromboxane *A*_2_ and adenosine diphosphate; which are potent platelet aggregators [18]. As these activated platelets enter areas of low ESS downstream, they accumulate due to delayed flow, which may trigger a coagulation cascade (Figure 1). Low ESS also leads to a reduction in nitric oxide (NO) production by the endothelium. NO plays an important role in maintaining the normal vascular tone and has anti-inflammatory and antithrombotic properties [25]. The cumulative effect of the above biomechanical principles is thought to be a significant driver for the progression of ST and ISR in segments with low post-PCI FFR measurements.

### 2.3. Practicalities of Post-PCI FFR Measurements

**Figure 2:**
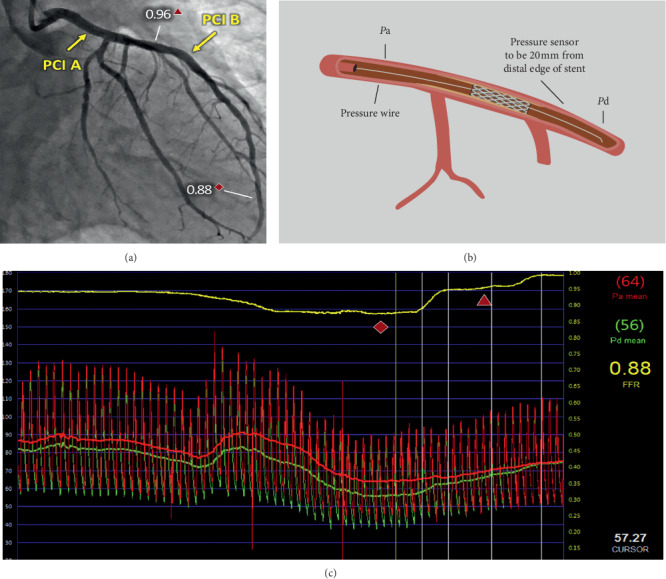
(a) Angiographic image of post-PCI FFR in the distal left main stem and mid-left anterior descending artery (b) Conceptual representation of post-PCI pressure measurements. FFR is calculated as a ratio of distal coronary pressure (*P*_d_) to aortic pressure (*P*_a_). FFR = *P*_d_/*P*_a_. (c) Pressure curve from post-PCI FFR measurement in the patient from part A, displaying pressure drops across the two stents. (a) and (c) are adapted from Ihdayhid et al. [30] with permission. Copyright © 2016, Elsevier.

Measurement of post-PCI FFR is similar to pre-PCI, although there are some subtle differences that require consideration. It should be noted that there is no requirement for patients to have pre-PCI FFR before performing post-PCI FFR assessment, although reimbursement of the pressure wire is often not possible in many healthcare systems for post-PCI FFR alone. Following angiographically satisfactory stent deployment, coronary pressure measurements are carried out at baseline and following induction of maximum hyperemia. Similar to pre-PCI FFR, the pressure sensor guidewire should be inserted and pressures equalized, with the wire then advanced distal to the stented segment [26]. Care has to be taken when manipulating the wire through the stent, to ensure the pressure sensor is not damaged on protruding struts. In pre-PCI FFR, the pressure wire is usually advanced 20–30 mm distal to the target lesion [27]. However, there appears a lack of consensus on the location of the pressure wire in post-PCI measurements. While the majority of studies do not report the exact location of the distal pressure segment. Li et al. placed the pressure wire 10 mm distal to the stent edge [7]. Meanwhile, the FFR-Search study measured ∼20 mm distal to the stent edge [28]. These differing definitions have potential to impact the FFR thresholds reported in the literature, with a lower FFR value being obtained the more distal the wire is placed [29]. Maximal hyperemia is achieved with intravenous adenosine infusion (140 mcg/kg per minute) or intracoronary boluses. FFR is then calculated as a ratio of distal coronary pressure (*P*_d_) to aortic pressure (*P*_a_) as shown in Figure 2. Pressure wire pullback is then completed to verify equal pressure signals from the wire and guiding catheter and to ensure the absence of signal drift [31].

Traditionally, the requirement of multiple wires for pressure gradient assessment has limited the use of FFR. However, newly developed pressure wires can be utilized as workhorse wires allowing for a streamlined process by reducing the number of wire changes required. The reduced procedural time and complexity with newer generation pressure wires should promote greater uptake of pre- and post-PCI FFR.

### 2.4. Interpretation of Suboptimal Post-PCI FFR

**Figure 3:**
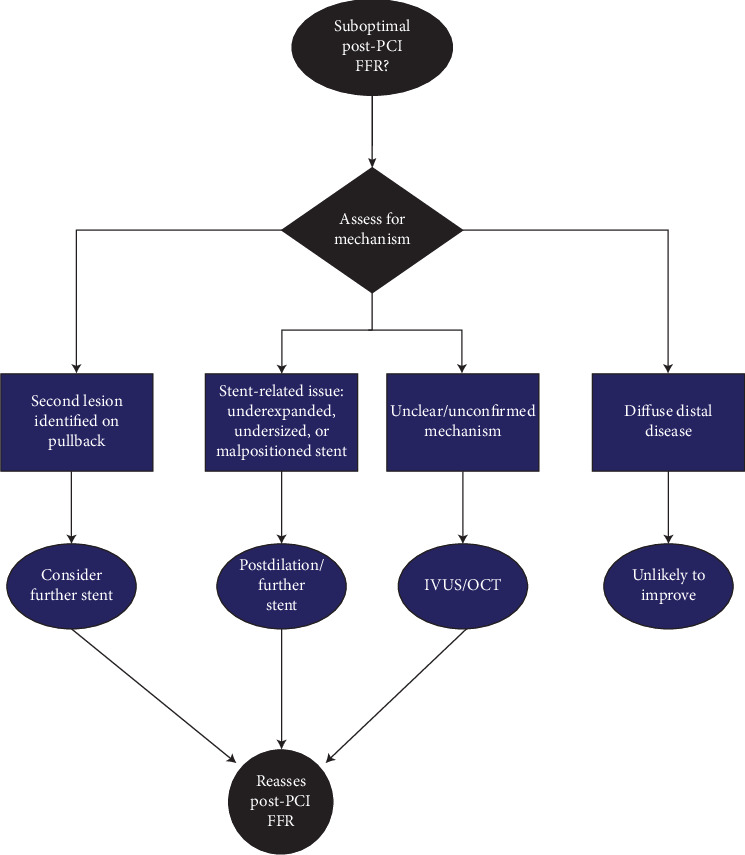
A simple schematic approach of interpretation of post-PCI FFR.

A substantial proportion of patients have reduced post-PCI FFR measurements, which may alert clinicians to the requirement of further action. It has been shown that the use of post-PCI FFR leads to additional intervention in 20% of lesions deemed angiographically satisfactory [8]. In this large prospective cohort, further intervention resulted in increment of FFR from 0.78 ± 0.07 to 0.87 ± 0.05 (*p* < 0.0001), leaving only 9% of lesions with persistent ischaemia [8]. An improvement in the post-PCI FFR does appear to translate into an improved clinical outcome [8, 32]. A simple framework to approaching suboptimal post-PCI FFR values is shown in Figure 3.

In patients with reduced post-PCI FFR, the operator should endeavor to exclude stent under-expansion, malapposition, and residual functionally significant lesions within the target vessel. Manual pullback can assist and should be performed to localize the area of pressure drop. On pullback, if a second lesion is localized or the stent appears undersized, further stenting or aggressive postdilatation may be considered. If manual pullback is unable to localize an area of pressure drop, the pressure transducer can also be positioned just distal and proximal to the edges of the stents to establish the pressure gradient over the stented segment. In the presence of a significant trans-stent gradient, further postdilation is advisable [10]. If there are still concerns regarding stent deployment or the exact mechanism remains unconfirmed, imaging through IVUS or OCT can be utilized to provide detailed visualization and precise quantification of expansion and plaque shift/protrusion [14, 17, 27].

It is also common to encounter diffuse disease in the vessel of interest that is not immediately evident. Diffusely atherosclerotic residual disease will cause a continuous pressure decline along the length of the vessel on manual pullback [33]. However, diffuse disease throughout the vessel is typically not amenable to further intervention, as there is no discrete lesion amenable to stenting [32]. Wolfrum et al. used OCT guidance to optimize the final FFR result and found 23% of stented segments were not amenable to PCI optimization and the FFR remained suboptimal regardless, usually secondary to diffuse distal disease [14].

### 2.5. FFR for Side Branch Assessment in Bifurcation Lesions

FFR can be particularly useful in guiding PCI of coronary bifurcation lesions. Current guidelines support the treatment of bifurcation lesions using a provisional strategy of stenting the main vessel (MV), followed by subsequent consideration of intervention to the jailed side-branch (SB) [34]. It is well recognized that angiographic assessment of the jailed SB is unreliable with only a small fraction (<30%) of angiographically severe jailed SB stenoses being FFR significant [35, 36]. There are both anatomical and physiological mechanisms to account for this observation. The degree of SB luminal narrowing following MV stenting is often the result of geometric carinal shift rather than plaque redistribution [37]. The mechanical forces contributing to carinal shift typically occur in a single direction, making the stenosis even more eccentric and therefore exaggerating angiographic severity of the jailed SB [37]. Physiologically, the trans-stenotic pressure gradient across a lesion and therefore FFR is highly dependent on the area of myocardium subtended [38, 39]. This explains the observation that, for a given stenosis and lesion length, lesions in major epicardial arteries tend to have lower FFR values than side-branch vessels by virtue of the degree of myocardium supplied [38].

**Figure 4:**
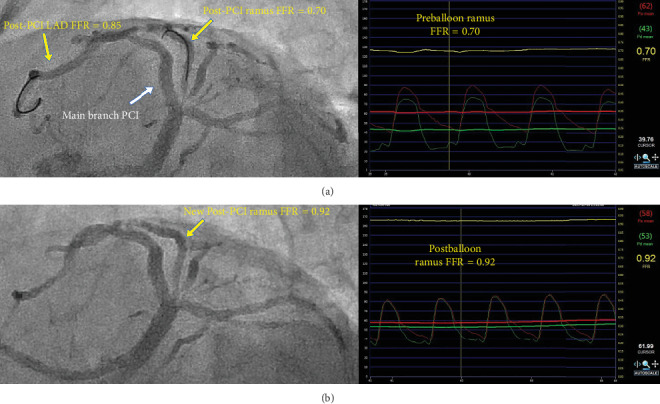
Use of post-PCI FFR for side branch assessment and optimization. (a) Suboptimal post-PCI FFR in the ramus (side branch) following bifurcation PCI, warranting further intervention. The pressure curve from this FFR measurement can be seen on the right. (b) Following kissing balloon dilation, post-PCI FFR in the ramus is improved.

Therefore, the functional evaluation of jailed side-branches should be considered in the context of the size of the side-branch and degree myocardial territory supplied. When clinically appropriate, FFR assessment of the jailed SB can reduce unnecessary complex SB intervention including the use of a second stent and reduce the incidence of MV restenosis [36, 40]. In the minority of patients with FFR significant jailed side branch stenoses, 93% of such lesions can be adequately treated with kissing balloon dilatation to achieve an FFR of ≥0.75 [41], as shown in Figure 4.

There are several technical issues to consider when performing an FFR of a jailed SB following MV stenting. The pressure wire is not as steerable as a standard guidewire and therefore passing the pressure wire through the MV struts is more challenging with rates of failure between 5 and 10% [35, 40]. This can be facilitated by kissing balloon inflation, proximal optimization technique in the MV, or engaging the SB with a standard guidewire and exchanging it for a pressure wire through a microcatheter [34, 42]. Finally, when interpreting the results of the SB ostial FFR, it is important to appreciate both upstream and downstream diseases, as this can influence FFR measurements and may act as to confound decision relating to SB stenting.

## 3. When Might Post-PCI FFR Not Be Reliable?

### 3.1. Long and Ultralong DES

Previous studies evaluating post-PCI FFR have predominantly demonstrated its prognostic value for clinical outcomes in an average stent length of <30 mm [7, 8, 11]. Baranauskas et al., however, suggested that this did not translate to patients with diffuse long segment coronary artery lesions requiring long (30–49 mm) or ultralong DES (≥50 mm). In this single-centre prospective study including 74 patients who received long or ultra-long DES, FFR >0.90 immediately after PCI was achieved only in 28.4% (21/74) of patients, of whom only two had received ultralong DES [43]. At 9-month follow-up, 61 patients had FFR measured; 23% of patients had an FFR >0.90, restenosis rate was 15.1% by functional assessment, and target lesion revascularization occurred in 8.1% of patients [43]. The rate of achieving FFR >0.90 immediately after PCI and at 9-month follow-up is considerably lower than that reported in the previous studies on shorter stents, suggesting that it is more challenging to achieve satisfactory post-PCI FFR values in patients treated with long or ultra-long DES. One possible mechanism is that patients requiring ultralong stents have a substantial burden of residual diffuse disease. Accordingly, the investigators observed an increased gradient in the distal unstented vessel in patients with reduced post-PCI FFR.

Reduced vascular compliance in long DES is another mechanism for a persistent post-PCI FFR gradient. Healthy coronary vessels and the microcirculation are able to regulate coronary flow even in the presence of atheroma, aiming to balance the coronary flow with myocardial oxygen requirements. Endothelial dysfunction is an accepted phenomenon secondary to mechanical injury of the vessel immediately after stent deployment [44]. Therefore, it is expected that longer stents will cause a larger area of damage, augmenting the process of vascular dysfunction [45]. This leads to a reduction in the endothelial release of vasoactive substances in the stented segment of a long DES. Normal vessel vasomotion is further limited by the larger metallic frame of a long DES producing a greater radial force resistive to changes in vessel diameter. These mechanisms provide a further explanation for lower post-PCI FFR values in long DES.

### 3.2. Microvascular Damage

The use of FFR in culprit vessels of patients with acute coronary syndrome (ACS) remains controversial [31]. Following myocardial infarction (MI), the microvasculature can be either transiently or permanently damaged. This limits microvascular vasodilatory capacity and maximal achievable hyperemic flow, leading to falsely negative FFR values [46]. This is particularly evident in patients with acute STEMI, where FFR of the culprit vessel was found to be unreliable and falsely elevated immediately following primary PCI compared with repeat measurements at 6-months (FFR 0.94 vs. 0.88; *p*=0.006) [47]. Higher post-PCI FFR values in STEMI patients compared with stable angina patients have been reported previously (FFR 0.95 vs. 0.90; *p*=0.002), despite similar intravascular ultrasound parameters [48]. Thus, FFR values are not valid in culprit vessel ACS, and the performance of FFR in this setting may significantly underestimate coronary lesion severity.

**Figure 5:**
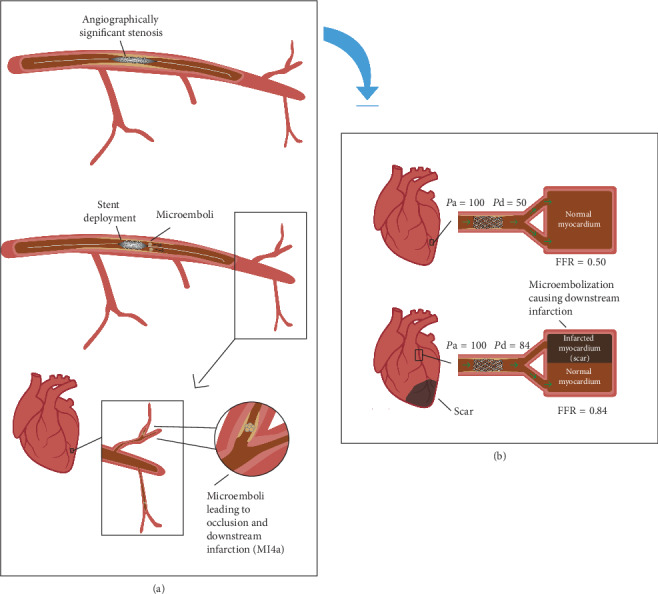
Illustration of falsely elevated FFR value in the setting of MI4a: (a) Depiction of downstream infarction due to MI4a. FFR calculation through a patient with a normal heart and no evidence of downstream flow limitation. (b) Falsely elevated FFR values may result secondary to MI4a. A falsely elevated FFR value in the setting of MI4A. MI4a limits the maximal achievable hyperemic flow and hence leads to a falsely elevated FFR value.

Similarly, microvascular dysfunction may also occur following PCI in stable patients, due to PCI-related myocardial infarction and microvascular injury (MI4a). This has been found to occur in approximately 30% of patients undergoing PCI for stable angina [49, 50]. Consequently, microvascular resistance is higher and coronary blood flow blunted, leading to a smaller pressure drop and falsely elevating FFR [51] (as shown in Figure 5). Hoole et al. demonstrated that in patients with normal microvascular function, PCI resulted in a significant increase in microvascular resistance in nontarget vessels, with resultant decreased coronary flow reserve and increased FFR values (FFR 0.79 vs. 0.81; *p* < 0.01) [51]. This study included 48% of patients who had MI4a following the procedure. At present, there are no studies that assess whether MI4a impacts post-PCI FFR in patients with stable angina and this remains an area of open research. Certainly, post-PCI FFR should not be performed in the culprit vessel of patients presenting with ACS, as these patients are known to have significant microvascular disruption that affects the validity of FFR assessment [52].

### 3.3. Current Evidence for Post-PCI FFR

There is growing evidence over the past two decades supporting the use of post-PCI FFR as a predictor of future clinical outcomes (Table 1). Studies have showed that a suboptimal post-PCI FFR correlates with a significantly higher rate of MACE and/or TVF [7–12, 15, 28, 32, 53–59]. One of the first studies to validate the concept of physiologically optimized PCI was Pijls et al. [11]. Here, the investigators studied 750 patients with post-PCI FFR following BMS. After 6 months, patients with a post-PCI FFR >0.95, FFR between 0.90 and 0.95, and FFR <0.90 had MACE rates of 4.9%, 6.2% and 29.5%, respectively. Both post-PCI FFR and stent length were shown to independent predictors of MACE. Ito et al.'s study was one of the earlier studies to explore FFR post-DES implantation including nonculprit ACS lesions [15]. Receiver operating characteristic curves were used to determine the post-PCI FFR cutoff of 0.90 in this cohort. The association between reduced post-PCI FFR with increased residual plaque volume and rates of MACE was similarly confirmed in this patient population.

Lee et al. showed that a post-DES FFR ≥0.84 was sufficient to lower the risk of TVF (2.6% vs. 9.1%, hazard ratio 3.37, *p*=0.006) [57]. Percentage FFR increase was also found to be a useful marker of procedural success, as patients with a low increase in %FFR (≤15%) were found to be at a higher risk of TVF (9.2% vs. 3%, hazard ratio 3.61, *p*=0.003). The FFR-search study released prospective registry data on 1000 consecutive patients that intriguingly failed to show a relationship between post-PCI FFR and 30-day clinical outcomes [28]. This study included culprit vessel ACS patients which is significant, given the known alterations in flow dynamics after MI. Nonetheless, these findings are not entirely unexpected, given that clinically significant ISR will typically require several months to develop. Long-term results of this study should follow and will be of interest.

Recently, post-PCI non-hyperemic indices were evaluated for the first time [59]. Here, both post-PCI FFR ≤0.86 (23% vs 17%, *p*=0.02) and non-hyperemic *P*_d_/*P*_a_ ≤ 0.96 (24% vs. 15%, *p* < 0.001) were associated with an increased risk of MACE at 30-month follow up. These provisional data suggest that non-hyperemic indices may also confer the same diagnostic information as FFR when interrogating immediate procedural results. While a pre-PCI FFR is not necessitated for post-PCI measurement, a greater improvement in FFR following PCI is also significantly associated with reduced rates of target vessel revascularization (*p*=0.01) [60].

### 3.4. Comparing Clinical Outcomes following Optimization with Post-PCI FFR vs OCT/IVUS

However, the use of IVUS, OCT, and post-PCI FFR when compared with angiographically guided PCI has been shown to reduce the rates of MACE [7, 61, 62]. There are not any studies that directly compare clinical outcomes between post-PCI FFR and intravascular imaging following PCI. The FORZA trial was a 1 : 1 randomized study that did compare OCT- and FFR-based assessment of intermediate severity lesions and consequent PCI optimization [63]. However, the results may not be generalizable beyond the context of the trial, due to the imbalance in routine post-PCI assessment between modalities (61% in FFR vs. 76% in OCT group, *p*=0.0017) [64]. Thus, it remains very unclear on whether FFR or intravascular imaging is the more suitable form of assessment following PCI.

Many interventionalists frequently utilize FFR before PCI, and this means that the upfront cost of the wire has already been absorbed. It therefore seems logical to reuse this wire following intervention, as an objective “free” method to assess the quality of the intervention. Use of the pressure wire in this setting may indeed act as a gatekeeper for use of additional costly intravascular imaging catheters, which are probably more likely to identify the actual reason for the suboptimal FFR method. As technology evolves and FFR starts to become available from imaging methods alone (e.g., angio-FFR), we may find physiology playing an increasing role following intervention.

### 3.5. Post-FFR Values as a Risk Continuum

As can be seen in Table 1, a broad range of post-PCI FFR cutoffs have been reported in studies to be associated with future outcomes. However, it appears more intuitive that post-PCI FFR should be viewed as a continuum for risk stratification. There is certainly no consensus over a single “best” FFR cutoff that predicts the risk of future repeat intervention. Initial studies appeared to report higher cutoffs in the range of 0.90–0.95. As the concept of using post-PCI pressure indices was in its infancy, studies often utilized low-risk lesions with limited residual disease in the target vessel [9, 10, 53]. In such patients, a higher post-PCI FFR value would be expected and hence higher cutoffs. Cutoffs have also been impacted by the transition from BMS to DES in the last decade, leading to lower rates of TVF and stent-related complications. Moreover, some studies reported pressure indices after stent insertion guided by IVUS or OCT, which would also contribute to higher FFR values [12, 15]. Perhaps, the reduction in cutoffs in newer studies is also partly indicative of post-PCI FFR being used in more complex lesion subsets [8, 28].

Results from the DKCRUSH VII Registry Study revealed that LAD lesions were predictive of a suboptimal post-PCI FFR in DES [7]. The concept of LAD/vessel specific post-PCI FFR cutoffs was introduced. A post-PCI FFR >0.91 in the LAD was found to have lower rates of TVF at 1 year (*p* < 0.001). Hwang et al. further explored this concept of physiological optimization specific to the target vessel (604 LAD and 232 non-LAD PCI's) [58]. It was found that the optimal cutoffs for post-PCI FFR in the LAD and non-LAD were 0.82 and 0.88, respectively.

Finally, the large heterogeneity between inclusion and exclusion criteria also contributes to the wide range of cutoffs. While there is no definitive post-PCI FFR cutoff value, post-PCI FFR should be used as a continuous function that directly relates to risk of future adverse clinical outcomes, with a lower value corresponding to a higher long-term risk.

## 4. Conclusion

There is considerable evidence that there is a clear association between post-PCI FFR values and long-term angiographic and clinical outcomes in patients with stable angina or nonculprit vessel MI. A suboptimal FFR following stent insertion warrants further assessment of the target vessel, with the aim of identifying the factors that may lead to future repeat revascularization. Large prospective trials are now required to cement the validity of post-PCI FFR and will help clinicians determine an optimal post-PCI FFR outcome.

## Figures and Tables

**Table 1:** Summary of trial data including FFR cutoffs and clinical and stent outcomes.

Study	Sample Size, *n*	Indication for PCI	FFR cut-off	Follow-up period, months	Primary endpoint	Main findings
Bech et al. [9]	58	Stable angina	≥0.9	24	MACE (death, MI, CABG, repeat PTCA, unstable angina)	Post-PCI FFR ≥0.9 is associated with significantly reduced MACE.
Pijls et al. [11]	750	ACS and stable angina	≥0.95	6	MACE (death, MI, CABG, TVR)	Post-PCI FFR >0.95 group had a MACE rate of 4.9%. Post-PCI FFR 0.9–0.95 had a MACE rate of 6.2%. Post-PCI FFR <0.80 group had a MACE rate of 29.5%. Post-PCI FFR was found to be an independent predictor of clinical outcomes.
Klauss et al. [53]	119	Stable angina	≥0.95	6	MACE (death, MI, TVR)	Post-PCI FFR >0.95 was associated with significantly less cardiac events.
Leesar et al. [10]	66	Stable angina	≥0.96	24	MACE (cardiac death, MI, TLR)	Post-PCI FFR ≥0.96 was associated with significantly lower MACE.
Nam et al. [54]	80	Stable angina and ACS	>0.9	12	MACE (death, MI, TVR)	The rate of MACE in the high FFR group (>0.9) was 2.5% compared with 12.5% in the low post-PCI FFR group (≤0.9).
Ito et al. [15]	97	Stable and nonculprit ACS	>0.9	17.8	MACE (cardiac death, MI, stent thrombosis, TVR)	The MACE rate was lower in patients with higher post-PCI FFR values. Optimal FFR threshold was found to be 0.9. Reduced FFR post-PCI was associated with higher residual plaque volume on IVUS.
Doh et al. [55]	107	Stable angina and nonculprit ACS	≥0.89	36	TVF (death and MI attributed to target vessel, TVR)	Patients with a post-PCI FFR ≥0.89 had significantly reduced TVF.
Reith et al. [12]	66	Stable angina	>0.905	20	MACE (death, MI, TLR)	The MACE rate was significantly lower in patients with post-PCI FFR 0.905. There was a fairly strong linear relationship between area stenosis on OCT and post-PCI FFR.
Agarwal et al. [8]	574	ACS and stable angina	>0.86	31	MACE (death, MI and TVR)	Patients who achieved a post-PCI FFR >0.86 had significantly lower MACE.
Li et al. [7]	1476	Stable and unstable angina	>0.88	36	TVF (cardiac death, target vessel MI, TVR)	Post-PCI FFR ≤0.88 strongly correlated with TVF. Disease in the LAD was an independent predictor of impaired FFR post-PCI. In LAD lesions, a post-PCI FFR >0.905 had lower rates of TVF at 1 year.
Piroth et al. [56]	639	Stable angina and stabilized ACS	≥0.92	24	TVF (death and MI attributed to target vessel, TVR)	Post-PCI FFR ≥0.92 was associated with a significantly reduced rate of TVF.
Lee et al. [57]	621	Stable angina and nonculprit ACS	≥0.84	24	TVF (cardiac death, target vessel MI, TVR)	Post-PCI FFR ≥0.84 was associated with a significantly lower risk of TVF.
Azzalini et al. [32]	65	Stable and unstable angina	≥0.9	12	MACE (cardiac death, MI, TVR, further angina)	The MACE rate was significantly lower in post-PCI FFR ≥0.9.
Hwang et al. [58]	835	Stable angina and nonculprit ACS	>0.84	24	TVF (cardiac death, target vessel MI, TVR)	Post-PCI FFR >0.84 had significantly reduced rates of TVF. The optimal cutoff value of post-PCI FFR in the LAD and non-LAD were 0.82 and 0.88, respectively.
Van bommel et al. [28]	1000	Stable angina and ACS	>0.9	1	MACE (cardiac death, MI, TVR)	Post-PCI FFR did not correlate with clinical outcomes at 30 days.
Hakeem et al. [59]	574	ACS and stable angina	>0.86	30	MACE (cardiac death, MI, TVR)	Post-PCI FFR ≤0.86 was predictive of a significantly increased risk of MACE. Nonhyperemic *P*_d_/*P*_a_ ≤ 0.96 was also predictive of MACE.

ACS = acute coronary syndrome. FFR = fractional flow reserve. MACE = major adverse cardiac events. MI = myocardial infarction. PCI = percutaneous intervention TVF = target vessel failure. TVR = target vessel revascularization.
